# Loop-Mediated Isothermal Amplification (LAMP) Assessment for Malaria Diagnosis in Equatorial Guinea

**DOI:** 10.3390/pathogens15080872

**Published:** 2026-08-20

**Authors:** Alexandra Martín-Ramírez, Teodora Mikumu Alogo, Victoria Palacios, Monserrat Kobe Elonga, Victoria Mangue, Irene Molina-de la Fuente, Vicenta González Mora, Marta Lanza-Suárez, Ana Rodríguez-Galet, Matilde Riloha Rivas, Policarpo Ncogo, Agustín Benito, José Miguel Rubio, Elizabeth Nyakarungu, Pedro Berzosa

**Affiliations:** 1 Malaria and Emerging Parasites Laboratory, National Center for Microbiology, Institute of Health Carlos III, 28222 Madrid, Spain; 2 Centro de Investigación Biomédica en Red de Enfermedades Infecciosas, Instituto de Salud Carlos III, 28029 Madrid, Spain; 3 Baney Research Laboratory, Baney P.O. Box 338, Equatorial Guinea; 4 Malariology Unit, Institute of Tropical Medicine of Antwerp, 2000 Antwerp, Belgium; 5 National Center of Tropical Medicine, Institute of Health Carlos III, 28029 Madrid, Spain; 6 National Malaria Programme, Ministry of Health and Social Welfare, Government of the Republic of Equatorial Guinea, Malabo, Equatorial Guinea; 7 Spanish State Foundation, Health, Children and Social Welfare, 28029 Madrid, Spain

**Keywords:** *Plasmodium falciparum*, PCR, malaria endemic area, molecular diagnosis, LAMP

## Abstract

Malaria diagnosis in Equatorial Guinea (EG) is based on conventional microscopy, sometimes combined with rapid diagnostic tests (RDTs). However, some laboratories, such as the Baney Research Laboratory (BRL) have implemented molecular technology for the diagnosis of infectious diseases. This study aimed to evaluate, for the first time in EG, a LAMP method for malaria diagnosis, comparing its performance with conventional microscopy performed in EG and with LAMP and PCR diagnostic methods performed at the National Centre of Tropical Medicine (NCTM), Spain. A total of 178 blood samples (including *P. falciparum* samples and malaria-negative controls) were evaluated using Dual-LAMP-Pspp and Nested-PCR-Pf in Equatorial Guinea. Dual-LAMP-Pspp performed at the BRL showed sensitivity and specificity values of 97.28% and 100%, respectively, for malaria diagnosis, with no differences between colorimetric and fluorescence-based result readings. Meanwhile, Nested-PCR-Pf showed a sensitivity of 87.07% and a specificity of 100%. Comparison of parasite densities determined by Dual-LAMP-Pspp and conventional microscopy showed a significant moderate negative correlation (correlation coefficient = −0.584). The comparison of Dual-LAMP-Pspp results between BRL and NCTM showed a kappa coefficient of 0.93, whereas the kappa coefficient of the Nested-PCR-Pf assay was 0.70. These findings indicate that Dual-LAMP-Pspp and Nested-PCR-Pf are suitable methods for malaria diagnosis in EG. However, the Dual-LAMP-Pspp assay was easy to perform and interpret and provided results in a shorter time than Nested-PCR-Pf.

## 1. Introduction

Malaria is a tropical and subtropical mosquito-borne disease caused by *Plasmodium* parasites [1]. An estimated 282 million cases were reported in 2024, representing an increase of 9 million cases compared with 2023 [2]. The WHO African Region bears the heaviest burden of mortality, accounting for 95% of malaria deaths worldwide [2,3] with *Plasmodium falciparum* causing most cases, and children and pregnant women being the most vulnerable populations [3,4].

Equatorial Guinea (EG) is located in West-Central Africa. It is divided into a Continental Region and an Insular Region (Bioko Island, where the capital city, Malabo, is located; and Anobón, Elobey Grande, Elobey Chico, and Corisco) [5]. The latest World Malaria Report [2] estimated approximately 431,000 malaria cases in 2024 in EG. The progress made in reducing malaria case incidence in 2024 compared to 2015 was less than the expected target (a decrease between 25% and 70%) [2]. Thus, it continues to be a public health problem and remains a significant cause of death, especially in children under five years of age [6]. The government of EG is implementing a strategy for malaria elimination throughout the country with different measures [7]. One of the fundamental strategies in malaria control is the proper management of patients with effective antimalarial drugs after the diagnosis of *Plasmodium* infection [8,9].

Malaria diagnosis is based on microscopic examination of Giemsa-stained thick and thin blood smears, often combined with rapid diagnostic tests (RDTs) [2]. Conventional microscopy remains the gold standard for diagnosing malaria [1]. It can differentiate *Plasmodium* species and quantify parasitemia [1]; however, it is time consuming, requires expert microscopists, and has limited sensitivity compared to other diagnostic techniques, such as molecular methods [10,11,12,13]. RDTs have been an important addition to microscopy for malaria diagnosis [14,15]. They are rapid, able to produce results in 15 to 20 min, easy to perform, and relatively affordable [10,16]. Nevertheless, this technique has several limitations, including lower sensitivity for species other than *P. falciparum*, the inability to quantify parasite density or assess treatment response, and the occurrence of false-negative results, which are becoming increasingly common in some countries due to parasites with *pfhrp2* gene deletions [10,15,16]. Additionally, when the goal is to interrupt transmission or eliminate malaria, all infections must be detected, including asymptomatic infections, which are usually of low density [17,18]. Conventional microscopy and RDTs generally detect high-density infections in resource-limited settings. However, they fail to detect a substantial proportion of infections that might be subpatent but remain infectious to mosquitoes [19]. Molecular methods are more sensitive and specific than conventional methods [18,20] and can detect submicroscopic infections [13,21]. PCR techniques are the most widespread molecular methods. However, they require sophisticated equipment, specific purification procedures, and a cold chain for reagent maintenance, which makes their use very limited in many endemic areas [22,23]. Loop-mediated isothermal amplification (LAMP) was first described in 2000 by Notomi et al. [24] as a new molecular method that amplifies DNA with high specificity, efficiency, and rapidity under isothermal conditions.

Several features make the LAMP technique a more versatile method than PCR and allow it to be applied in low-resource malaria-endemic areas [20,25,26,27]. These features include: (i) a stable reaction temperature of 60–65 °C, eliminating the need to use a thermocycler to adjust the temperature and time, and requiring only a water bath or heating block, which allows LAMP reactions to be performed directly in the field without specialized equipment [28,29,30]; (ii) high tolerance for inhibitors of the Bst polymerase enzyme [31,32], which allows for simple nucleic acid extraction processes; (iii) high sensitivity and specificity values, similar to those of molecular methods such as PCR [22,24]; (iv) a shorter turnaround time, with the entire amplification process completed in one hour or less [33]; and (v) multiple methods for the detection of the amplified products, including gel electrophoresis, turbidity, fluorescence, color change, or systems based on lateral flow assays [29,30].

All these features make LAMP an appealing method for detecting malaria cases in endemic countries [34,35], suitable for use in screening by national malaria control programs [25,34].

Malaria diagnosis in EG is mainly based on thick blood smear microscopy, sometimes combined with RDTs, in most regions of the country [12].

This study aimed to assess, for the first time in Equatorial Guinea, a LAMP method for the diagnosis of malaria and to compare its performance with that of LAMP and PCR methods performed in a reference laboratory in a non-endemic area.

## 2. Materials and Methods

### 2.1. Study Area and Blood Sample Collection

**Figure 1:**
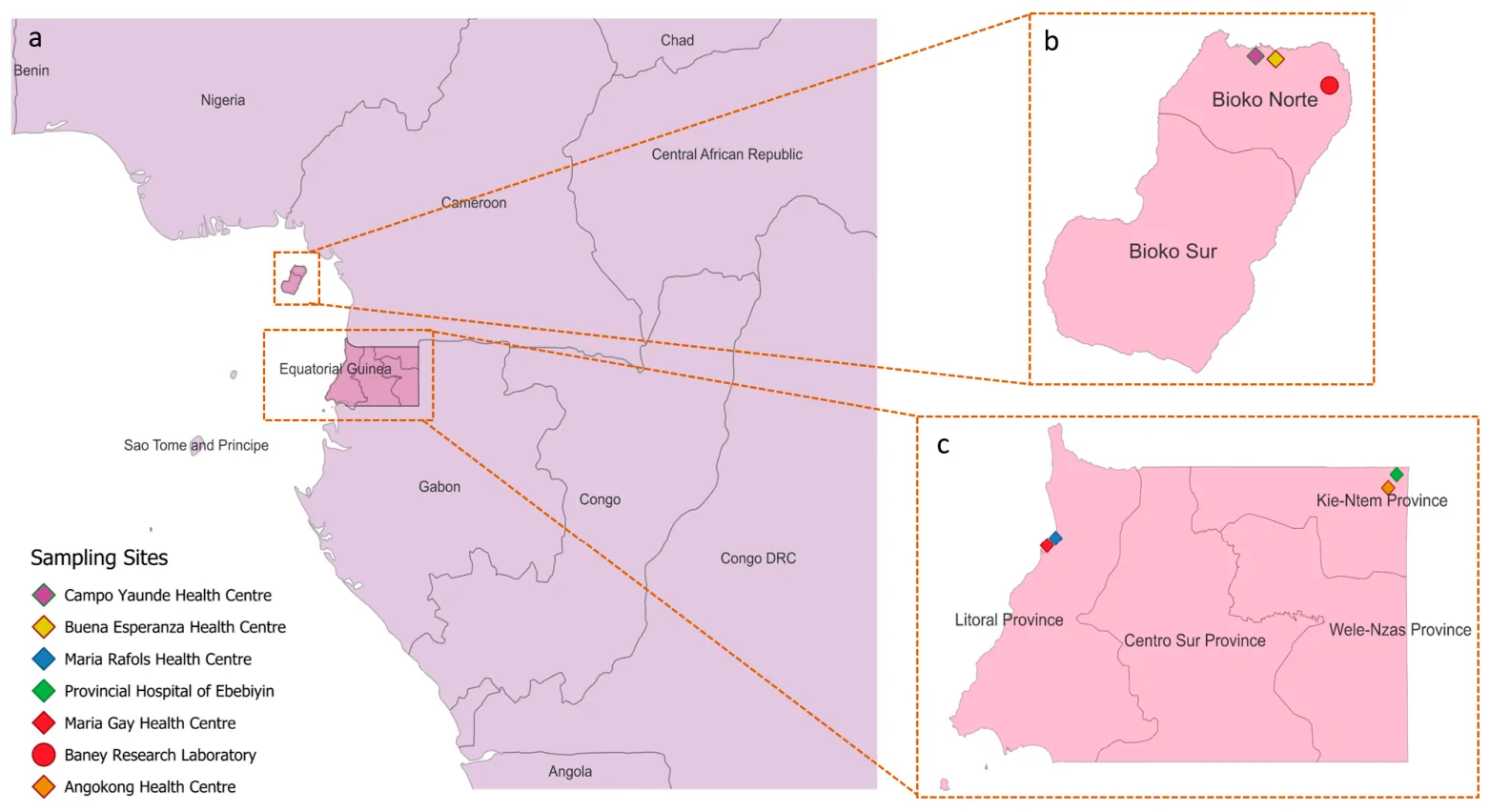
Map of Equatorial Guinea in the African continent (**a**), Bioko Island (**b**) and Continental Region (**c**). The triangles indicate the health centers where sampling took place (**b**,**c**), while the red dot indicates the laboratory where samples were analyzed and negative controls were obtained (**b**).

The study included a total of 178 blood samples. Malaria-positive blood samples (147) were collected from symptomatic patients in six health centers in the Continental Region and on Bioko Island in EG (Ebebiyín: Angokong Health Center and Provincial Hospital of Ebebiyín; Bata: Maria Gay Health Center and Maria Rafols Health Center; and Malabo: Campo Yaunde Health Center and Buena Esperanza Health Center) (Figure 1).

Capillary blood samples obtained by finger prick were collected to perform malaria diagnosis. Field thick blood smear microscopy was performed immediately, while two drops of blood were spotted on Whatman 903™ paper (GE Healthcare Bio-Sciences Corp., Piscataway, NJ, USA), dried, and individually inserted into a zip-lock bag with silica gel. One blood spot on Whatman paper per sample was transferred to the National Center of Tropical Medicine (NCTM), Institute of Health Carlos III, Madrid (Spain), for molecular analysis, while the other blood spot was sent to the Baney Research Laboratory (BRL), Malabo, Equatorial Guinea. All dried blood spots (DBSs) were stored at −20 °C until analysis. Samples were collected from March to June 2024. Demographic data, such as sex and age, were recorded for each sample.

Thirty-one samples were obtained from individuals without malaria, who were identified as PCR-negative at the Baney Research Laboratory. These samples were the negative controls for the study.

**Figure 2:**
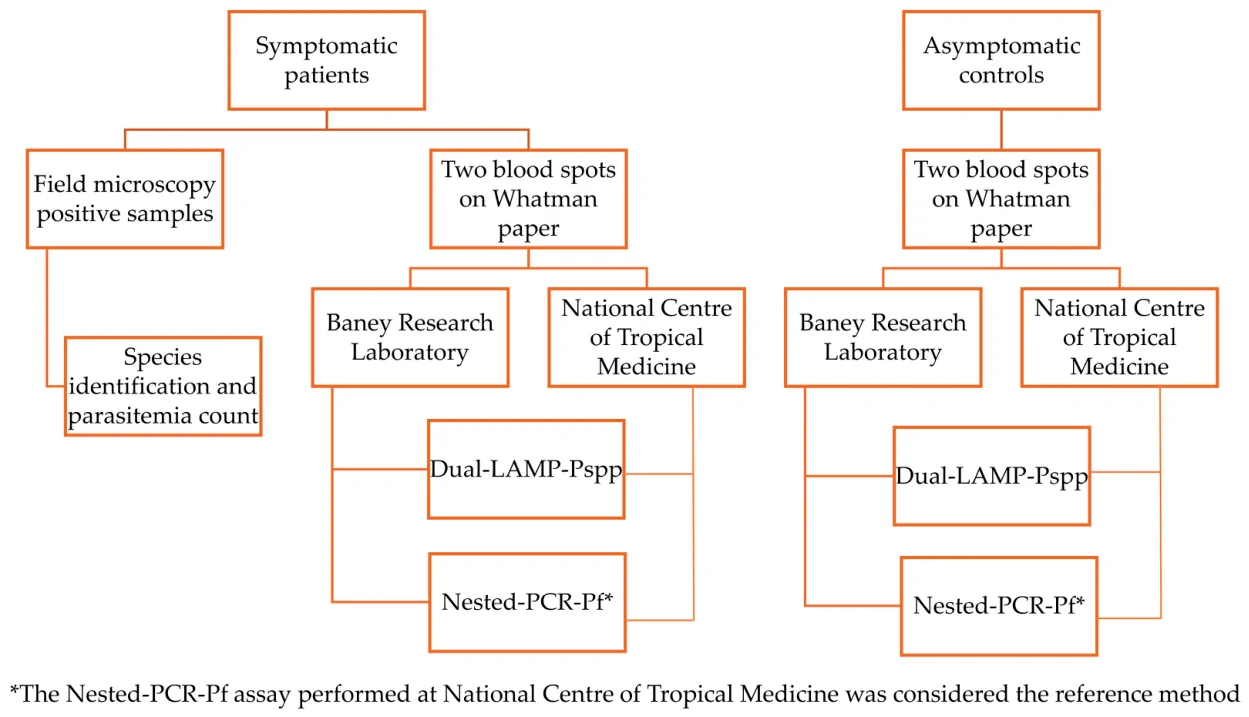
Flowchart of the employed samples, techniques, and performance laboratories.

LAMP and PCR malaria diagnostic tests (Dual-LAMP-Pspp and Nested-PCR-Pf) were performed in parallel in both BRL and NCTM laboratories (Figure 2).

### 2.2. Conventional Microscopy

Thick blood films were stained with 10% Giemsa for 15 min in staining jars. All slides were read by two trained microscopists under 100× magnification with immersion oil in each health center. In case of any discrepancy between the two trained microscopists, the blood smear was reviewed by a third expert microscopist. Parasitemia was quantified in parasites/µL following WHO guidelines [36] and the presence of gametocytes was recorded.

### 2.3. DNA Extraction

DNA was extracted from the DBS samples using the saponin–Chelex method with minor modifications adapted to our laboratory [37]. Briefly, a 5 mm diameter filter paper disc containing 10 μL of blood was incubated with 1 mL of 0.5% saponin in 1× PBS at 37 °C for 1 h or at 4 °C overnight. The saponin solution was then removed, and the disc was washed with 1 mL of 1× PBS. Subsequently, 200 μL of 5% Chelex-100, preheated to 98 °C for 10 min, was added to each sample, and the samples were incubated at 98 °C for 10 min. Samples were centrifuged at 14,000 rpm for 2 min, and the supernatant was carefully transferred to a new tube without disturbing the Chelex pellet. Following a second centrifugation at 14,000 rpm for 2 min, the supernatant containing the extracted DNA was transferred to a screw-cap tube. The extracted DNA was used for PCR and LAMP reactions, and the remaining DNA was stored at −20 °C.

### 2.4. Nested-PCR

PCR was performed as described by Snounou et al. [38] with some modifications adapted to our laboratory. It was selected as the reference molecular method based on its demonstrated high sensitivity for *Plasmodium* detection and its extensive use and established performance in our laboratory. PCR reactions were based on the sequence of the small subunit ribosomal RNA genes. The first PCR used two genus-specific primers, while the product obtained was used for a second amplification cycle using species-specific primers for *P. falciparum* (Nested-PCR-Pf) [38,39,40]. Briefly, the first round of PCR used the primer pair PLU5 (5′-CCTGTTGTTGCCTTAAACTTC-3′) and PLU6 (5′-TTAAAATTGCAGTTAAAACG-3′), while second-round PCR used the primer pair FAL1 (5′-TTAAACTGGTTTGGGAAAACCAAATATATT-3′) and FAL2 (5′-ACACAATGAACTCAATCATGACTACCC-3′). The first-round PCR reaction was performed in a 20 μL final volume containing 1× reaction buffer, 0.125 mM dNTPs, 0.125 μM of each primer, and 0.4 U of Taq DNA polymerase. For the initial PCR, 2 μL of extracted DNA was added to the reaction mixture.

For the nested PCR, 2 μL of the first-round PCR product was used as the template. The reaction was performed in a 25 μL final volume containing 1× reaction buffer, 0.2 mM dNTPs, 0.32 μM of each primer, and 0.5 U of Taq DNA polymerase. The thermocycling conditions for the first PCR included initial denaturation at 95 °C for 5 min, followed by 30 cycles of 53 °C for 2 min, 72 °C for 2 min, and 94 °C for 1 min. This was followed by an additional annealing step at 53 °C for 2 min and a final extension at 72 °C for 10 min. The second-round PCR was performed with an initial denaturation at 95 °C for 5 min, followed by 35 cycles of denaturation at 95 °C for 1 min, annealing at 58 °C for 45 s, and extension at 72 °C for 1 min, followed by a final extension at 72 °C for 10 min.

The presence of amplification was detected by agarose gel electrophoresis of the second PCR, with a fragment size of 205 bp for *P. falciparum* amplified fragments. Positive, negative, and no-DNA controls were included in all runs.

### 2.5. LAMP for Malaria Diagnosis

The genus-specific *Plasmodium* spp. LAMP (Dual-LAMP-Pspp) described by Martin-Ramirez et al. [22] was performed on all samples. Briefly, the reaction mix was composed of WarmStart^®^ Colorimetric LAMP 2X Master Mix buffer (New England Biolabs, Ipswich, MA, USA) with phenol red dye for colorimetric detection, and a set of six specific primers for *Plasmodium* spp. (F3: 5′-GTATCAATCGAGTTTCTGACC-3′; B3c: 5′-CTTGTCACTACCTCTCTTCT-3′; FIP: 5′-TCGAACTCTAATTCCCCGTTACCTATCAGCTTTTGATGTTAGGGT-3′; BIP: 5′-CGGAGAGGGAGCCTGAGAAATAGAATTGGGTAATTTACGCG-3′; LPF: 5′-CGTCATAGCCATGTTAGGCC-3′; LPB: 5′-AGCTACCACATCTAAGGAAGGCAG-3′ [41], 1× EvaGreen dye for real-time fluorescence detection, and 5 μL of DNA in a final volume of 25 μL. Dual-LAMP-Pspp reactions were performed in a CFX96 Touch Real-Time PCR Detection System (Bio-Rad Laboratories, Inc., Hercules, CA, USA) in BRL and in a Rotor-Gene^®^ thermocycler (QIAGEN^®^ GmbH, Hilden, Germany) in NCTM. Reactions were incubated for 30 min at 65 °C, with green channel fluorescence measured every minute, followed by 5 min at 80 °C for inactivation.

Results were read using: (i) colorimetric detection, in which the reaction changed from pink to yellow when amplification occurred, and (ii) fluorescence reading in the thermal cycler. Fluorescence results were expressed as the time to amplification (Ta), defined as the time at which the fluorescence signal increased above the baseline level established from the negative controls and no-template control (NTC), indicating a positive result. Samples that did not show fluorescence above this threshold were considered negative. For colorimetric results, ambiguous color differences were resolved by considering orange results as positive and reddish results as negative.

A positive control, a negative control, and a no-DNA extraction control were included in all batches from the DNA purification step. Samples showing discordant results compared with the reference method were re-tested in duplicate.

### 2.6. Operational Characteristics

Estimated costs and time requirements were calculated. The estimated costs included the reagents for a single assay, excluding expenses related to controls, equipment, or personnel. Personnel costs were estimated based on the time required to perform each technique. Turnaround time was defined as the period from the start of nucleic acid extraction to the completion and interpretation of the results, whereas hands-on time referred to the actual time spent by laboratory staff performing the different steps of the procedures.

### 2.7. Statistical Analysis

The overall concordance rate (percentage of true-positive and true-negative results among the total number of results) and kappa value (k) were determined to measure the degree of agreement between BRL and NCTM for the results of the two diagnostic tests, Dual-LAMP-Pspp and Nested-PCR.

Test accuracy measures, including clinical sensitivity, specificity, positive (PPV) and negative (NPV) predictive value, with their respective 95% confidence intervals, were calculated for Dual-LAMP-Pspp in BRL and NCTM using EPI DAT (3.1) software [42]. Nested-PCR-Pf performed at NCTM was considered the reference method.

Parasite density values obtained by thick blood smear microscopy were compared with Ta values obtained with Dual-LAMP-Pspp from samples that provided a positive result with both techniques. Spearman’s correlation coefficient was calculated using R version 4.4.2, after performing the Shapiro–Wilk normality test.

## 3. Results

### 3.1. Comparative Performance of Microscopy, PCR and LAMP in Malaria-Endemic and Non-Endemic Settings

The mean age of malaria-positive patients was years and 10 months, and 44.9% were female. All microscopy-positive samples were identified as *P. falciparum* infections. The geometric mean parasite density among microscopy-positive samples was 33,358 parasites/µL (range 23–686,567 parasites/µL), and gametocytes were detected in 4.76% of samples (7/147).

**Figure 3:**
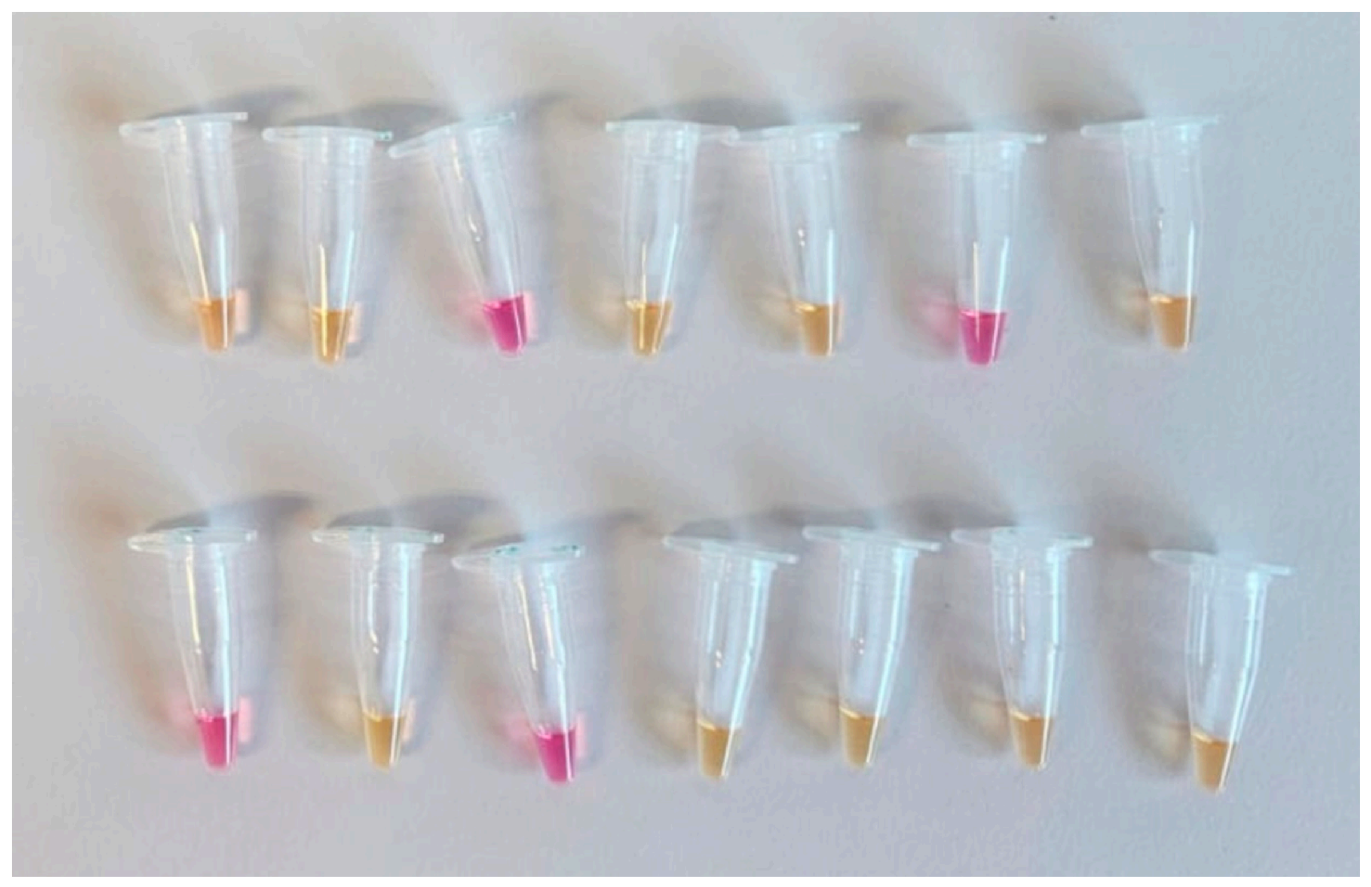
Colorimetric detection of *Plasmodium* spp. by the Dual-LAMP-Pspp assay. Yellow tubes indicate positive results, whereas pink tubes indicate negative results.

The Dual-LAMP-Pspp assay detected *Plasmodium* spp. in 143 of the 147 samples that tested positive by conventional microscopy in the malaria-endemic setting (BRL) (Table 1 and Supplementary Table S1), with Ta values ranging from 6.01 to 15.41 in the fluorescence readouts (Supplementary Table S1 and Figure S1). When the assay was performed using blood spots in the non-endemic setting (at the National Center of Tropical Medicine), 145 of the 147 samples identified as malaria-positive by field microscopy were also positive by Dual-LAMP-Pspp (Table 2). All samples from asymptomatic controls yielded negative results with the Dual-LAMP-Pspp assay, regardless of the setting in which the assay was performed. No differences were observed between the colorimetric (Figure 3) and fluorescence-based readings of the Dual-LAMP-Pspp assay.

When compared with the reference method, Dual-LAMP-Pspp showed a sensitivity of 97.28% in the malaria-endemic setting laboratory and 98.64% in the reference laboratory, while specificity was 100% in both settings (Table 1 and Table 2).

Nested-PCR-Pf detected *P. falciparum* in 128 of the 147 microscopy-positive samples in the malaria-endemic setting, while all 31 samples from asymptomatic controls yielded negative results (Table 1 and Supplementary Table S1). In the reference laboratory, Nested-PCR-Pf identified 147 positive and 31 negative samples (Table 2). The sensitivity of Nested-PCR-Pf in the malaria-endemic setting was 87.07%, whereas its specificity was 100% (Table 1).

**Figure 4:**
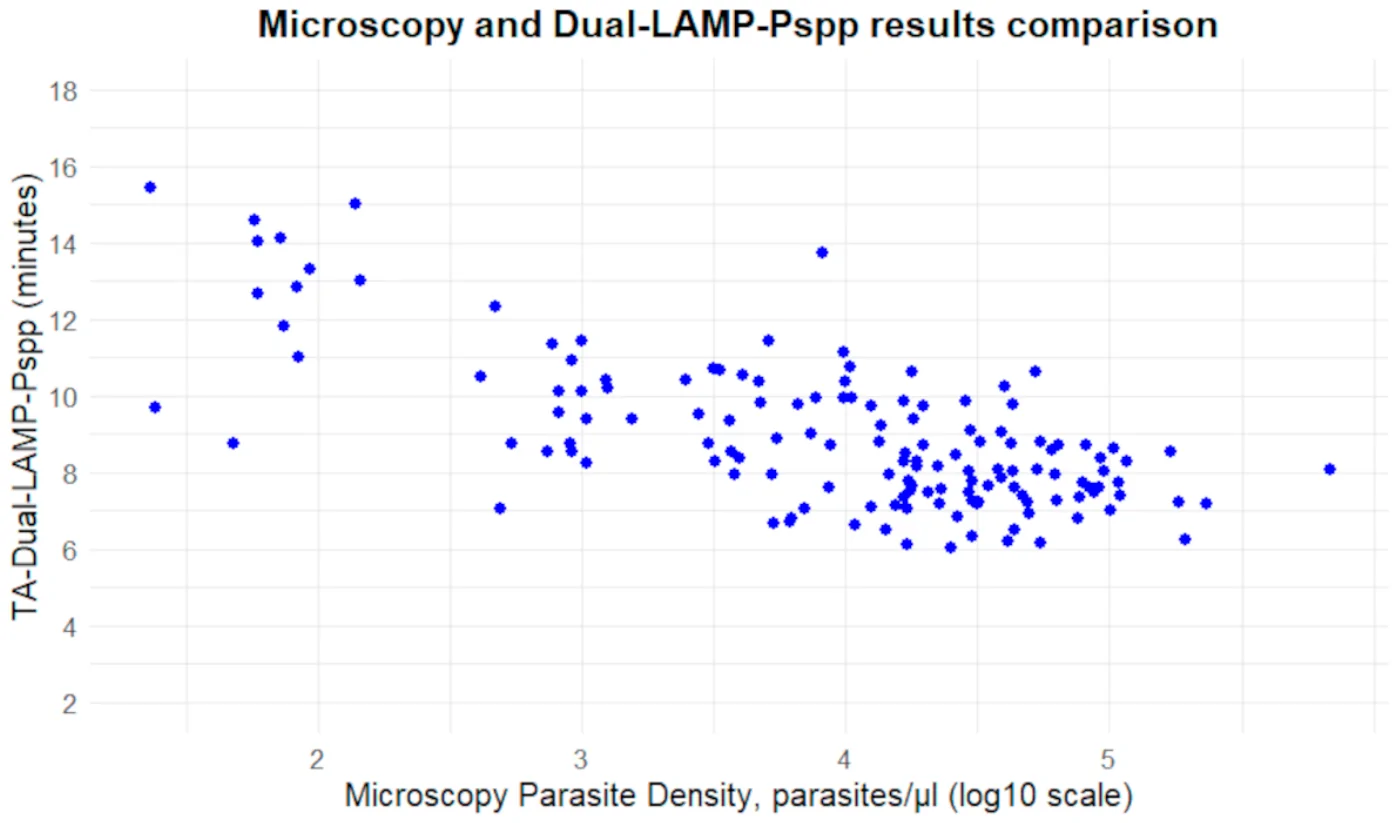
Correlation between parasite density (log_10_ parasites/μL), measured by thick blood smear microscopy, and TA values (minutes), measured by Dual-LAMP-Pspp, among samples positive by both diagnostic methods (*n* = 143).

A comparison between parasite density determined by microscopy and Ta values obtained with Dual-LAMP-Pspp was performed for the 143 samples that were positive by both methods. A statistically significant moderate negative correlation was observed between parasite density and Ta values (*p* < 0.001; correlation coefficient = −0.584) (Figure 4).

### 3.2. Comparison of LAMP and PCR Assays Between Malaria-Endemic and Non-Endemic Settings

The results obtained with the Dual-LAMP-Pspp and Nested-PCR-Pf assays performed in BRL and NCTM were compared. The concordance of the Dual-LAMP-Pspp assay between the two settings was 97.75% (kappa coefficient = 0.93, 95% CI: 0.86–0.99), whereas the concordance of the Nested-PCR-Pf assay was 89.33% (kappa coefficient = 0.70, 95% CI: 0.58–0.82).

### 3.3. Operational Characteristics

The total turnaround time for diagnosis using the Dual-LAMP-Pspp assay, from sample receipt to result interpretation, was estimated at approximately two hours. This included one hour for sample processing and DNA purification, 30 min for master mix preparation and LAMP setup, and 30 min for the LAMP reaction and result interpretation. The estimated hands-on time was approximately one hour.

The total turnaround time for diagnosis using the Nested-PCR-Pf assay was estimated at approximately 8 h and 30 min. This included one hour for sample processing and DNA purification, 30 min for preparation of the first master mix, approximately three hours for the first PCR, 30 min for preparation of the second master mix, 2 h and 30 min for the second PCR, 45 min for electrophoresis, and 15 min for result analysis. The estimated hands-on time was approximately 2 h and 30 min.

The estimated reagent cost per assay, excluding the costs associated with controls, equipment, and personnel, was €3.50 for the Dual-LAMP-Pspp assay and €1.40. for the Nested-PCR-Pf assay.

## 4. Discussion

Blood smear microscopy and RDTs are the conventional methods for malaria diagnosis [1]. Their sensitivity is lower than that of malaria molecular methods [18,20]; however, they are useful for detecting clinical malaria in endemic areas [25,27,43]. Nevertheless, in many endemic regions, microscopy quality assurance (QA) is not provided, or initial training is provided but not maintained, with the assumption that microscopists remain competent throughout their careers [15,44,45]. In addition, microscopy and RDTs are not always used in combination for malaria diagnosis. These situations may lead to misdiagnosis of malaria cases, which may affect the management of patients and impact malaria transmission [12]. In contrast, molecular methods have higher sensitivity than conventional methods such as microscopy and RDTs and are able to detect symptomatic and asymptomatic malaria cases; therefore, they may be particularly useful for malaria elimination strategies [18,34]. The most common molecular technique for pathogen detection is PCR, although it requires specific extraction procedures and costly and specific equipment, which limits its use in endemic areas [35]. On the other hand, LAMP works with simple DNA extraction procedures, such as the boil-and-spin method [46], and common laboratory equipment, such as a dry-block heater and water baths [41]. Its sensitivity has been shown to be higher than that of microscopy and RDT methods [20], and comparable to that of PCR methods for malaria diagnosis in several non-endemic and endemic countries [47,48]. However, one limitation of LAMP is the difficulty of incorporating multiple targets within the same reaction tube, such as an internal amplification control, which would help to distinguish true negative results from potential amplification failures and identify false-negative results.

Equatorial Guinea is a malaria-endemic country, with *P. falciparum* as the main malaria species [49,50]. The prevalence of *P. falciparum* malaria was reported as 10.3% and 46.5% in Bioko Island and the Continental Region, respectively [51]. Malaria diagnostic methods performed in EG vary across the country, with conventional microscopy and RDTs, alone or combined depending on the laboratory, as the primary techniques for malaria diagnosis. However, malaria microscopy quality assurance is not performed in most areas, and a high number of false-positive and false-negative results from microscopy have been reported [12], in addition to the false-negative results from RDTs [12,51]. Therefore, there is a need to implement molecular methods for malaria detection that can be useful as point-of-care techniques and in malaria control and elimination strategies, in line with the malaria elimination strategy being implemented throughout the country.

Our study showed high sensitivity and specificity for the Dual-LAMP-Pspp assay for malaria diagnosis, with a kappa value of 0.925, which highlights the high concordance between the Dual-LAMP-Pspp assay and the reference method, indicating almost perfect agreement. Only four false-negative results were observed (Supplementary Table S1), all of which were consistently negative when the samples were re-tested in duplicate. These discordant results were most likely associated with low parasitemia levels and the consequently lower sensitivity of the Dual-LAMP-Pspp assay compared with the reference method, rather than with technical errors during DNA purification or amplification. Previous reports in endemic areas have shown high concordance between LAMP and PCR methods for malaria diagnosis [20,47,52]. However, this is the first time that a LAMP technique has been evaluated for malaria diagnosis in EG. The colorimetric and fluorescence readouts did not differ, which implies greater versatility for this technique to be applied in different settings in malaria-endemic countries, with colorimetric readouts being particularly useful in areas where no specific equipment is available. The comparison of microscopy parasite density and Ta values obtained with Dual-LAMP-Pspp provided a significant but moderate negative correlation. LAMP assays have shown lower time-to-amplification (Ta) values with higher parasite counts in other studies [22,53]; however, the correlation obtained in our study was lower. Different factors may explain this, such as the auto-cycling displacement DNA synthesis characteristic of the LAMP technique, which produces large amounts of DNA; the Ta values obtained with Dual-LAMP-Pspp, which ranged from 6.01 to 15.41 min, although most positive samples showed low Ta values, with an average of 8.91 min; or potential errors in microscopy parasite counts. Consequently, the Dual-LAMP-Pspp assay is a highly useful technique for determining whether a sample is positive or negative but is not intended for quantifying parasitemia.

The Nested-PCR-Pf assay performed in BRL also showed high sensitivity and specificity (over 85%) and a kappa coefficient of 0.70, which indicates substantial agreement. The lower agreement between the Nested-PCR-Pf assays performed in BRL and NCTM was due to 19 false-negative results obtained in BRL (Supplementary Table S1). This could be attributed to operational challenges, potentially linked to electrophoresis procedures. Although electrophoresis is a common technique, performed in most molecular biology laboratories, the malaria-endemic laboratories that acquired molecular biology equipment during the COVID-19 pandemic, such as the BRL, are more accustomed to performing commercial real-time PCR (qPCR) assays than conventional molecular biology techniques such as nested PCR. Commercial qPCR assays are relatively easy to perform, with the main requirement being to mix the reagents and analyze the fluorescence curves, whereas in-house nested-PCR assays require two PCR steps in addition to the agarose gel electrophoresis, which may further increase the risk of contamination. These drawbacks may have contributed to the high rate of false-negative results obtained with the Nested-PCR-Pf assay in BRL, whose concordance with the same assay performed in the reference laboratory at NCTM was 89.33%. In contrast, the concordance of the Dual-LAMP-Pspp assay between the two laboratories (in malaria-endemic and non-endemic settings) was higher, at 97.75%, likely because the Dual-LAMP-Pspp assay is easy to perform and the results are easier to interpret.

Although the applications of these molecular assays (Dual-LAMP-Pspp and Nested-PCR-Pf) differ due to their distinct functions, they may serve complementary roles. The Dual-LAMP-Pspp assay could be applied in laboratories where malaria diagnosis is performed using conventional methods. The shortage of experienced microscopists, the absence of regular microscopy QA programs, and the lack of properly maintained microscopes or adequate reagents in certain laboratories in malaria-endemic countries limit diagnostic reliability and increase the rate of false-positive and false-negative results [12]. Dual-LAMP-Pspp could serve as a point-of-care assay for malaria diagnosis in these laboratories. In fact, the operational characteristics showed that the Dual-LAMP-Pspp assay is much faster than Nested-PCR-Pf, highlighting its potential for use as a point-of-care malaria diagnostic tool. However, Dual-LAMP-Pspp could be particularly useful in malaria control and elimination strategies to detect microscopic and submicroscopic infections throughout the country and in other malaria-endemic countries. A meta-analysis of the diagnostic accuracy of LAMP [34] found that LAMP-based assays are appropriate for detecting low-level malaria parasite infections in the field and could become a valuable tool for malaria control and elimination programs. The colorimetric readouts of the Dual-LAMP-Pspp assay should be used where no thermocycler equipment is available. However, if a thermocycler is available, both fluorescence and colorimetric readings may be used to support result interpretation, especially if there is uncertainty regarding color interpretation, due to the higher subjectivity of color interpretation [22]. In contrast, the application of the Nested-PCR-Pf assay would be more restricted to reference laboratories with sufficient experience in molecular biology techniques. This PCR may be useful in combination with other protocols that may be implemented in malaria-endemic countries, such as the study of *pfhrp2* and *pfhrp3* deletions to assess the prevalence of false-negative RDT results associated with these deletions. Nevertheless, the application of any of these PCR techniques in other laboratories would require training and an initial evaluation of the performance of these assays.

Some limitations of this study include the evaluation of both LAMP and PCR assays in *P. falciparum* samples, without including samples infected with non-falciparum species, and the evaluation of the Dual-LAMP-Pspp assay in only one laboratory in EG. A broader evaluation of this assay should include more field laboratories around the country and assess its performance using a water bath or a heating block. In addition, both molecular assays assessed in this study require a cold chain for reagent storage, a limitation that could be overcome with lyophilized reagents. Furthermore, another limitation may be the use of Nested-PCR for *P. falciparum* detection instead of quantitative real-time PCR (qPCR) as the reference method. Nested-PCR requires two successive rounds of amplification, increasing the overall processing time and the risk of carryover contamination. However, the lower cost of PCR reagents compared with those required for qPCR was an important consideration given the available resources. Finally, the number and composition of the positive and negative samples were determined by the availability of well-characterized clinical samples, with a relatively small number of negative controls and the inclusion of predominantly symptomatic patients in the positive cohort. Although these factors may limit the generalizability of the diagnostic performance estimates, all samples were thoroughly characterized using established reference methods.

## 5. Conclusions

Dual-LAMP-Pspp and Nested-PCR-Pf assays provided reliable results for malaria diagnosis at the Baney Research Laboratory in Equatorial Guinea. Both assays showed high concordance with the corresponding assays performed at the reference laboratory at the Institute of Health Carlos III. However, Dual-LAMP-Pspp was easier to perform and interpret and provided results in a shorter time than Nested-PCR-Pf. This assay could be applied to other laboratories in EG and other malaria-endemic countries as a point-of-care diagnostic tool or as part of malaria control and elimination programs.

## Figures and Tables

**Table 1:** Results obtained using conventional microscopy, Dual-LAMP-Pspp and Nested-PCR-Pf in the malaria-endemic setting (Baney Research Laboratory), together with the sensitivity, specificity, positive predictive value (PPV), negative predictive value (NPV), and kappa coefficient of Dual-LAMP-Pspp and Nested-PCR-Pf compared with Nested-PCR-Pf performed in National Center of Tropical Medicine.

	Thick Blood Smear Microscopy	Dual-LAMP-Pspp Colorimetric Reading	Dual-LAMP-Pspp Fluorescence Reading		Nested-PCR-Pf
Positive	147	143	143		128
Negative	NA	35	35		50
**Diagnostic method**	**Sensitivity (CI)**	**Specificity (CI)**	**PPV** **(CI)**	**NPV** **(CI)**	**Kappa** **Coefficient (CI)**
Dual-LAMP-Pspp	97.28% (94.31–100%)	100% (98.39–100%)	100% (99.65–100%)	88.57% (76.60–100%)	0.925 (0.85–0.99)
Nested-PCR-Pf	87.07% (81.31–92.84%)	100% (98.39–100%)	100% (99.61–100%)	62% (47.55–76.45%)	0.70 (0.58–0.82)

NA: not applicable. Conventional microscopy was not performed on asymptomatic negative controls. CI = 95% confidence interval.

**Table 2:** Results obtained using Dual-LAMP-Pspp and Nested-PCR-Pf in the reference laboratory (National Center of Tropical Medicine); together with the sensitivity, specificity, positive predictive value (PPV), negative predictive value (NPV), and kappa coefficient of the Dual-LAMP-Pspp assay compared with Nested-PCR-Pf.

		Dual-LAMP-Pspp Colorimetric Reading	Dual-LAMP-Pspp Fluorescence Reading		Nested-PCR-Pf
	Positive	145	145		147
	Negative	33	33		31
**Diagnostic method**	**Sensitivity (CI)**	**Specificity** **(CI)**	**PPV** **(CI)**	**NPV** **(CI)**	**Kappa** **Coefficient (CI)**
Dual-LAMP-Pspp	98.64% (96.43–100%)	100% (98.39–100%)	100% (99.66–100%)	93.94% (84.28–100%)	0.961 (0.91–1.00)

CI = 95% confidence interval.

## Data Availability

The original contributions presented in this study are included in the article/Supplementary Material. Further inquiries can be directed to the corresponding authors.

## Supplementary Materials

The following supporting information can be downloaded at: https://www.mdpi.com/article/10.3390/pathogens15080872/s1, Figure S1: Ta values of Dual-LAMP-Pspp in concordant and discordant samples; Table S1: Results of the 178 samples analyzed using Nested-PCR-Pf and Dual-LAMP-Pspp at the Baney Research Laboratory (Equatorial Guinea), compared with the reference method.

## Abbreviations

The following abbreviations are used in this manuscript:

BRL	Baney Research Laboratory
EQ	Equatorial Guinea
Dual-LAMP-Pspp	*Plasmodium* spp. LAMP assay
LAMP	Loop-mediated isothermal amplification
Nested-PCR-Pf	Specific *Plasmodium falciparum* Nested-PCR assay
NCTM	National Center of Tropical Medicine
RDT	Rapid Diagnostic Test
