# Supplementary material for: Loop-Mediated Isothermal Amplification (LAMP) Assessment for Malaria Diagnosis in Equatorial Guinea
Source: Pathogens. 2026 Aug 20;15(8):872. doi: 10.3390/pathogens15080872 (PMC13516477; doi:10.3390/pathogens15080872)
Supplement: Supplementary file 1 [file pathogens-15-00872-s001.zip › pathogens-4489630-supplementary.pdf]

Supplementary material

Table S1. Results of the 178 samples analyzed using Nested-PCR-Pf and Dual-LAMP-Pspp at the Baney Research Laboratory (Equatorial Guinea), compared with the reference method. Results highlighted in bold indicate discrepancies with the reference method.

Sample code	Nested-PCR-Pf at NCTM, Spain (reference method)	Nested-PCR-Pf at BRL, Equatorial Guinea	Dual-LAMP-Pspp, at BRL, Equatorial Guinea (color readouts) *	Dual-LAMP-Pspp, at BRL, Equatorial Guinea (fluorescence readouts, Ta minutes)
1	Positive	Positive	Y	7.2
2	Positive	Positive	Y	8.73
3	Positive	Positive	Y	7.64
4	Positive	Positive	Y	8.26
5	Positive	**Negative**	Y	11.83
6	Positive	Positive	Y	8.39
7	Positive	Positive	Y	8.44
8	Positive	Positive	Y	8.5
9	Positive	Positive	Y	7.79
10	Positive	Positive	Y	8.07
11	Positive	Positive	Y	8.8
12	Positive	Positive	Y	9.38
13	Positive	Positive	Y	8.09
14	Positive	Positive	Y	7.62
15	Positive	Positive	Y	10.75
16	Positive	Positive	Y	7.46
17	Positive	Positive	Y	8.01
18	Positive	Positive	Y	8.01
19	Positive	**Negative**	Y/O	14.01
20	Positive	Positive	Y	7.59
21	Positive	Positive	Y	7.76
22	Positive	Positive	Y	8.56
23	Positive	Positive	Y	8.74
24	Positive	Positive	Y	7.93
25	Positive	Positive	Y	7.24
26	Positive	Positive	Y	6.17
27	Positive	Positive	Y	6.80
28	Positive	**Negative**	Y	6.64
29	Positive	Positive	Y	6.19
30	Positive	Positive	Y	7.05
31	Positive	Positive	Y	7.10
32	Positive	Positive	Y	7.50
33	Positive	Positive	Y	6.12
34	Positive	**Negative**	Y	9.69
35	Positive	Positive	Y	6.48

36	Positive	Positive	Y	9.71
37	Positive	Positive	Y	6.93
38	Positive	Positive	Y	6.34
39	Positive	Positive	Y	6.01
40	Positive	Positive	Y	7.07
41	Positive	Positive	Y	7.05
42	Positive	Positive	Y	8.71
43	Positive	Positive	Y	6.68
44	Positive	Positive	Y	7.37
45	Positive	Positive	Y	6.83
46	Positive	Positive	Y	6.71
47	Positive	Positive	Y	8.53
48	Positive	Positive	Y	9.84
49	Positive	Positive	Y	8.08
50	Positive	Positive	Y	7.56
51	Positive	Positive	Y	7.95
52	Positive	Positive	Y	6.50
53	Positive	Positive	Y	7.17
54	Positive	Positive	Y	8.54
55	Positive	Positive	Y	7.66
56	Positive	Positive	Y	8.30
57	Positive	Positive	Y	8.01
58	Positive	Positive	Y	8.36
59	Positive	Positive	Y	8.16
60	Positive	**Negative**	Y	8.16
61	Positive	Positive	Y	9.41
62	Positive	Positive	Y	7.95
63	Positive	Positive	Y	7.41
64	Positive	Positive	Y	8.77
65	Positive	Positive	Y	7.21
66	Positive	Positive	Y	8.81
67	Positive	Positive	Y	7.48
68	Positive	Positive	Y	7.37
69	Positive	Positive	Y	7.12
70	Positive	Positive	Y	8.27
71	Positive	Positive	Y	7.28
72	Positive	Positive	Y	7.19
73	Positive	Positive	Y	7.27
74	Positive	Positive	Y	7.00
75	Positive	Positive	Y	6.25
76	Positive	Positive	Y	9.85
77	Positive	Positive	Y	10.1
78	Positive	Positive	Y	8.28
79	Positive	Positive	Y	10.67
80	Positive	Positive	Y	9.94
81	Positive	Positive	Y	11.02

82	Positive	Positive	Y	9.5
83	Positive	Positive	Y	9.02
84	Positive	Positive	Y	8.78
85	Positive	Positive	Y	8.3
86	Positive	**Negative**	Y	12.32
87	Positive	Positive	Y	9.78
88	Positive	**Negative**	**R/P**	**Negative**
89	Positive	Positive	Y	7.93
90	Positive	Positive	Y	7.22
91	Positive	Positive	Y	9.35
92	Positive	Positive	Y	9.22
93	Positive	**Negative**	**R/P**	**Negative**
94	Positive	Positive	Y	7.4
95	Positive	Positive	Y	7.49
96	Positive	**Negative**	O	14.1
97	Positive	Positive	Y	10.43
98	Positive	Positive	Y	8.63
99	Positive	Positive	Y	10.63
100	Positive	Positive	Y	7.6
101	Positive	Positive	Y	10.61
102	Positive	Positive	Y	9.38
103	Positive	**Negative**	**R**	**Negative**
104	Positive	Positive	Y	8.76
105	Positive	**Negative**	Y	13.73
106	Positive	Positive	Y	8.89
107	Positive	Positive	Y	8.71
108	Positive	**Negative**	Y	13.01
109	Positive	**Negative**	**R**	**Negative**
110	Positive	Positive	Y	8.7
111	Positive	Positive	Y	8.58
112	Positive	Positive	Y/O	11.36
113	Positive	Positive	Y	10.22
114	Positive	**Negative**	Y	12.65
115	Positive	Positive	Y	6.81
116	Positive	Positive	Y	7.86
117	Positive	Positive	Y	7.75
118	Positive	**Negative**	Y	10.49
119	Positive	Positive	Y	7.61
120	Positive	Positive	Y	9.54
121	Positive	Positive	Y	8.76
122	Positive	**Negative**	Y	13.29
123	Positive	Positive	Y	10.13
124	Positive	**Negative**	Y/O	12.84
125	Positive	Positive	Y	9.8
126	Positive	Positive	Y/O	14.56
127	Positive	Positive	Y	9.77

128	Positive	Positive	Y	9.72
129	Positive	Positive	Y	10.38
130	Positive	Positive	Y	15.01
131	Positive	Positive	Y	8.77
132	Positive	Positive	Y	10.69
133	Positive	Positive	Y	10.35
134	Positive	Positive	Y	10.43
135	Positive	**Negative**	O	15.41
136	Positive	Positive	Y	10.92
137	Positive	Positive	Y	9.94
138	Positive	Positive	Y	9.05
139	Positive	Positive	Y	11.43
140	Positive	Positive	Y	9.11
141	Positive	Positive	Y	10.52
142	Positive	**Negative**	Y	10.23
143	Positive	Positive	Y	8.52
144	Positive	Positive	Y	11.12
145	Positive	Positive	Y	11.43
146	Positive	Positive	Y	7.75
147	Positive	Positive	Y	9.94
148	Negative	Negative	P	Negative
149	Negative	Negative	P	Negative
150	Negative	Negative	P	Negative
151	Negative	Negative	P	Negative
152	Negative	Negative	P	Negative
153	Negative	Negative	P	Negative
154	Negative	Negative	P	Negative
155	Negative	Negative	P	Negative
156	Negative	Negative	P	Negative
157	Negative	Negative	P	Negative
158	Negative	Negative	P	Negative
159	Negative	Negative	P	Negative
160	Negative	Negative	P	Negative
161	Negative	Negative	P	Negative
162	Negative	Negative	P	Negative
163	Negative	Negative	P	Negative
164	Negative	Negative	P	Negative
165	Negative	Negative	P	Negative
166	Negative	Negative	P	Negative
167	Negative	Negative	P	Negative
168	Negative	Negative	P	Negative
169	Negative	Negative	P	Negative
170	Negative	Negative	P	Negative
171	Negative	Negative	P	Negative
172	Negative	Negative	P	Negative
173	Negative	Negative	P	Negative

174	Negative	Negative	P	Negative
175	Negative	Negative	P	Negative
176	Negative	Negative	P	Negative
177	Negative	Negative	P	Negative
178	Negative	Negative	P	Negative

*O = orange; P = pink; R = reddish; Y = yellow.

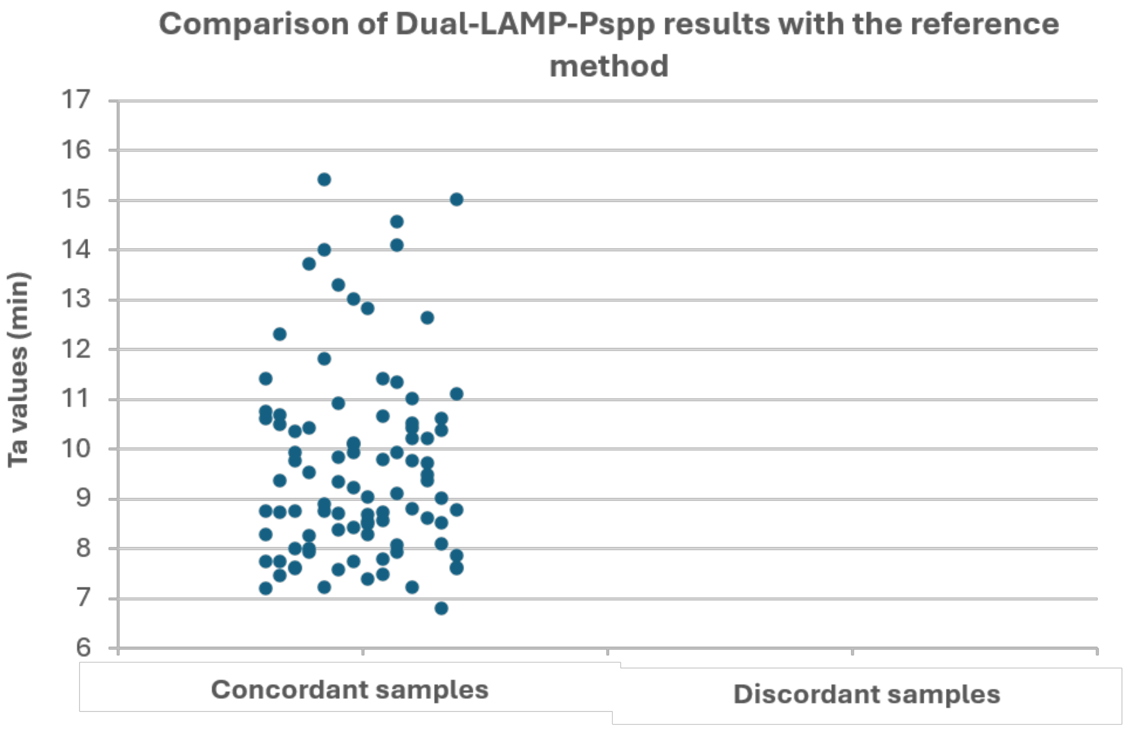

Figure S1: Ta values of Dual-LAMP-Pspp in concordant and discordant samples. Each dot represents an individual sample. Ta values are shown in minutes for samples with concordant and discordant results compared with the reference method.
